# A Rare Case with a Solitary Fibrous Tumour of the Colon and an Epithelioid Angiomyolipoma of the Kidney

**DOI:** 10.1155/2013/324538

**Published:** 2013-09-19

**Authors:** Thong Quang Pham, Shinsuke Aida, Ichiro Mori, Takeo Koshida, Takashi Ohigashi, Katsuyoshi Katase, Fumiyoshi Murakami, Thong Minh Tran, Robert Y. Osamura

**Affiliations:** ^1^Center for Diagnostic Pathology, Mita Hospital, International University of Health and Welfare, Tokyo 108-8329, Japan; ^2^Department of Pathology, Cho Ray Hospital, Ho Chi Minh City, Vietnam; ^3^Division of Urology, Mita Hospital, International University of Health and Welfare, Tokyo 108-8329, Japan; ^4^Women's Oncology Center, Sanno Medical Center, Tokyo 108-8329, Japan

## Abstract

Solitary fibrous tumour is a soft tissue tumour composed of a subset of fibroblast-like cells and frequently needs immunohistochemical staining for final diagnosis. Epithelioid angiomyolipoma is a variant of angiomyolipoma but characterized by the absence of both adipocytes and abnormal blood vessels. We introduce a very rare case with the combination of these two tumours. A Japanese female patient without significant symptom was hospitalized and operated due to multiple uterine leiomyomas. During the operation, the surgeons found another tumour attaching to serosa of sigmoid colon. This tumour was resected and interpreted as solitary fibrous tumour, suspicious of malignancy. After 13 months of treatment, she was hospitalized again due to hematuria. The doctors detected a tumour in her right kidney. After consultation, laparoscopic right nephrectomy was done. The pathological result of this tumour was epithelioid angiomyolipoma. This is the first report on this very rare combination of tumours with extensive immunohistochemical demonstration of both tumours. Hereby, we review clinical information and histopathological findings together with discussion on each tumour.

## 1. Introduction

Among the renal tumours, angiomyolipoma is widely known as a benign mesenchymal tumour composed of a variable proportion of adipose tissue, spindle and epithelioid smooth muscle cells, and abnormal thick-walled blood vessels [1]. However, one of its variants called epithelioid angiomyolipoma has some different features from usual angiomyolipoma. It is composed of purely epithelioid cells and characterized by the absence of both adipocytes and abnormal blood vessels. Epithelioid angiomyolipoma is potentially malignant because it can recur locally, metastasize, and cause death [2]. According to a study of 194 cases of renal angiomyolipoma, there are 15 cases (7.7%) of epithelioid angiomyolipoma [3].

Solitary fibrous tumour is a soft tissue neoplasm, formerly thought to be of mesothelial nature and limited to mesothelium-covered surfaces, and is now known to be composed of a subset of fibroblast-like cells and to be quite ubiquitous [4]. Extrapleural solitary fibrous tumours are uncommon. According to anatomic distribution of 298 solitary fibrous tumours of all sites, the tumours in internal abdomen accounted for 20% [5].

Renal epithelioid angiomyolipoma and intra-abdominal solitary fibrous tumour have some similar features which need differential diagnoses. Both of them are very uncommon. Here we report a very rare case of both renal epithelioid angiomyolipoma and intra-abdominal solitary fibrous tumour.

## 2. Case Presentation

A 54-year-old Japanese woman was hospitalized with many tumours in the uterus consistent with multiple leiomyomas and cysts in both ovaries. She had not had a significant symptom or history of disease. The operation was done in February 2012. However, when the patient was operated, the surgeons accidentally detected a tumour attaching to the serosa of sigmoid colon. The surgeons resected uterus, bilateral adnexa, and the unidentified tumour. All of specimens were sent to the diagnostic pathology center. Afterward, the diagnosis of specimen of uterus and bilateral adnexa was multiple leiomyomas of the uterus and endometriosis, hemorrhagic follicular cysts of both ovaries. Malignant findings were not seen in these lesions.

**Figure 1:**
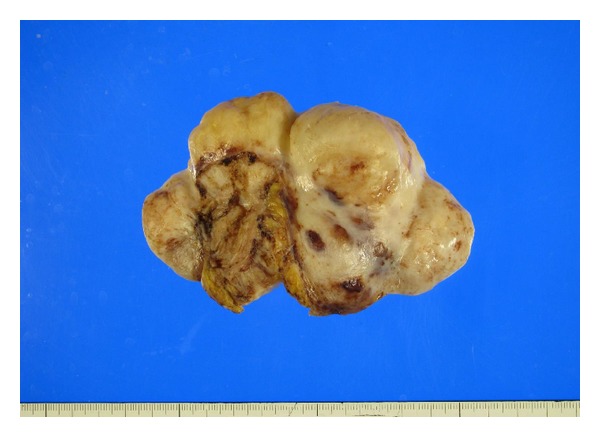
The solid tumour with multiple nodules attaching to serosa of sigmoid colon.

The incidentally identified tumour of the colon (Figure 1) was also examined. Grossly, the tumour was a yellow, solid mass measuring 7.5 × 6.0 × 5.0 cm. The tumour was composed of multiple nodules and well circumscribed.

**Figure 2:**
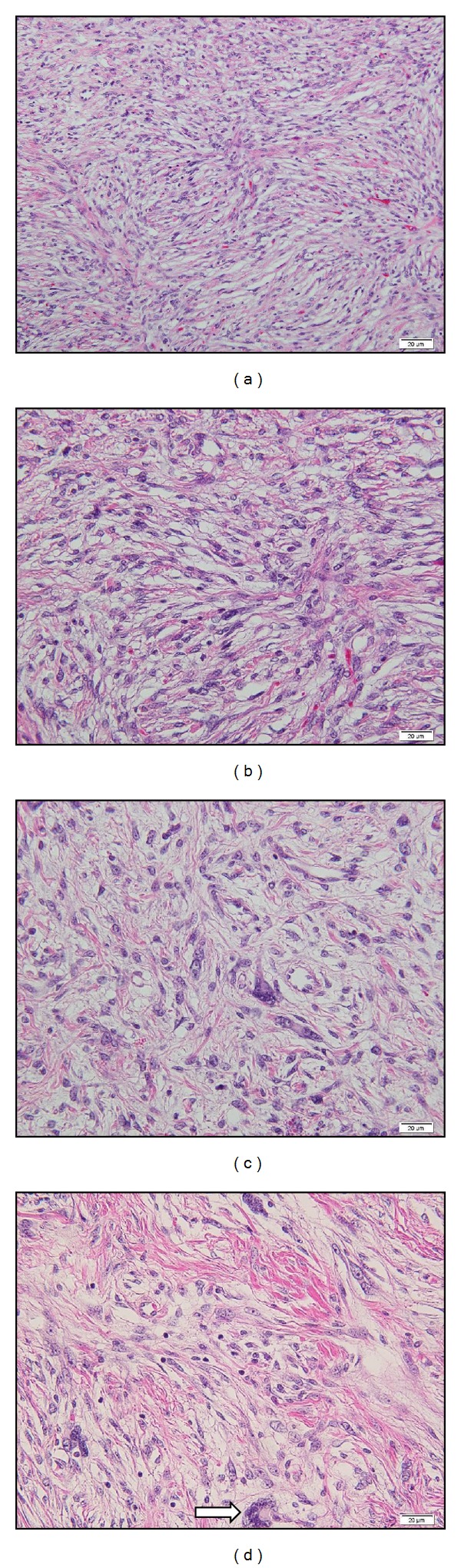
Light microscopic appearance of solitary fibrous tumor. (a) Low magnification and (b) high magnification show frequent storiform pattern in the tumor. (c), (d) Intermediate magnification reveals spindle cells and a few atypical plump spindle tumor cells. In (d), a multinucleated giant cell (⇒) is also seen.

**Figure 3:**
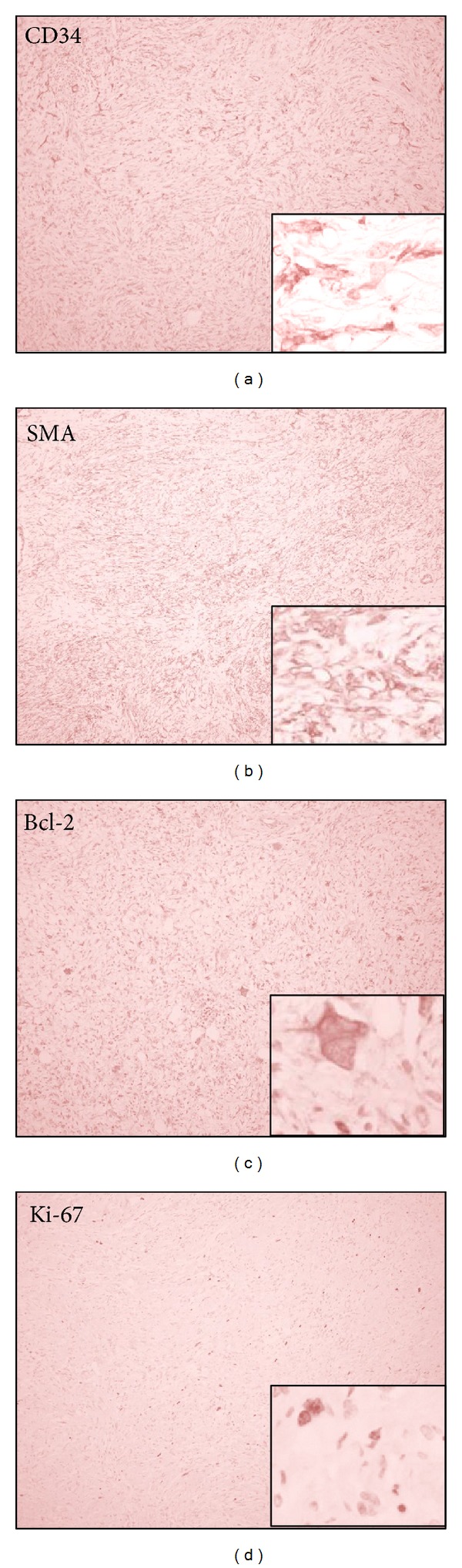
Immunohistochemical staining for the tumour attaching to serosa of sigmoid colon. CD34, Bcl-2, and SMA: positive for spindle cells. Desmin, 5100, C kit, and HMB 45: negative. Ki-67 labelling index: 10%.

Microscopically, the tumour was composed of alternating hypercellular and hypocellular areas. Most of the cells were spindle-shaped with indistinct cytoplasm and oval-shaped nuclei with dispersed chromatin. Some tumour cells were multinucleated or had large and hyperchromatic nuclei (Figure 2). Mitotic activity was low, about 1 mitosis per 10 high-power fields. The cellular pattern was not clear although there were some foci of storiform growth. Some vessels were branching. The thin parallel strands of collagen separated the tumour areas. By immunohistochemical staining, the tumour cells were positive for CD34, Bcl-2, and SMA and negative for desmin, S100, C-kit, and HMB-45. The Ki-67 labelling index was about 10% (Figure 3). These findings were interpreted as solitary fibrous tumour, suspicious for malignancy. The patient was treated by chemotherapy.

**Figure 4:**
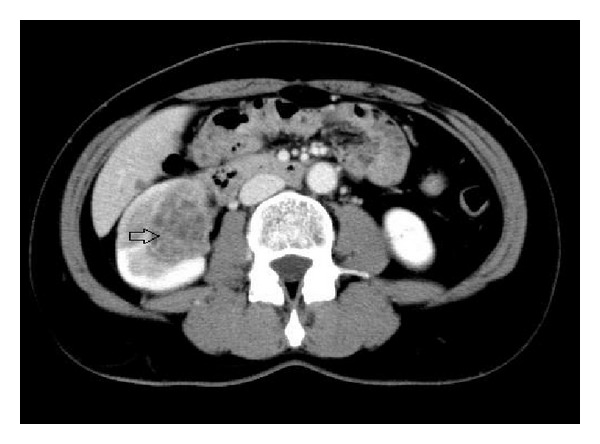
CT scan image of the abdomen showing the tumour (arrow) in the right kidney.

**Figure 5:**
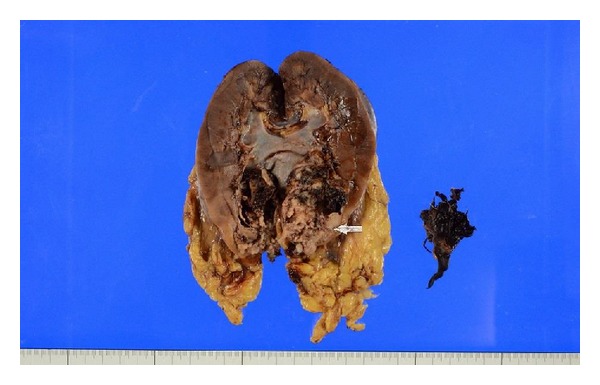
The tumour (arrow) in the right kidney after surgery.

Thirteen months after the operation, the patient returned to the hospital due to hematuria. By contrast enhanced computed tomography, a tumour in the lower portion of the right kidney was detected (Figure 4). After consultation, laparoscopic right nephrectomy was done. The weight of her right kidney was 228 grams. It was transferred to diagnostic pathology center for examination. Grossly, the tumour showed soft, yellow, and haemorrhaging mass measuring 5.0 × 3.0 × 2.5 cm (arrow). Border of the tumour was well defined but pushing and unencapsulated (Figure 5).

**Figure 6:**
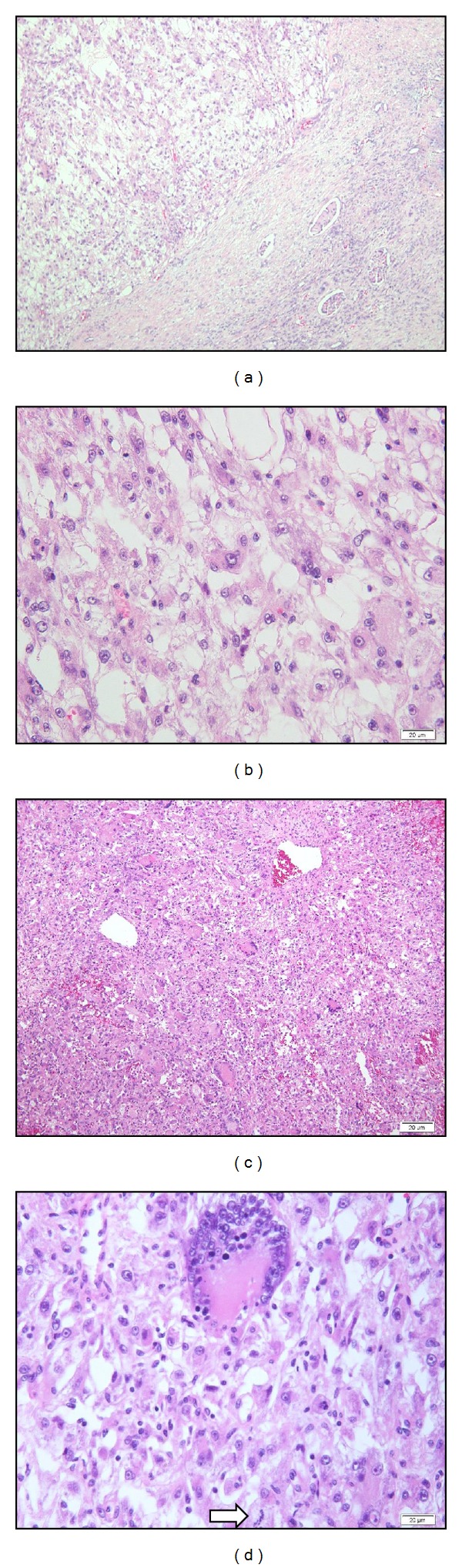
Light microscopic features of epithelioid angiomyolipoma. (a) low magnification and (b) high magnification of epithelioid features of the tumor cells. Adjacent renal tissue in (a). (c) Low magnification and (d) high magnification of the areas with mixture of epithelioid cells and multinucleated giant cells.

**Figure 7:**
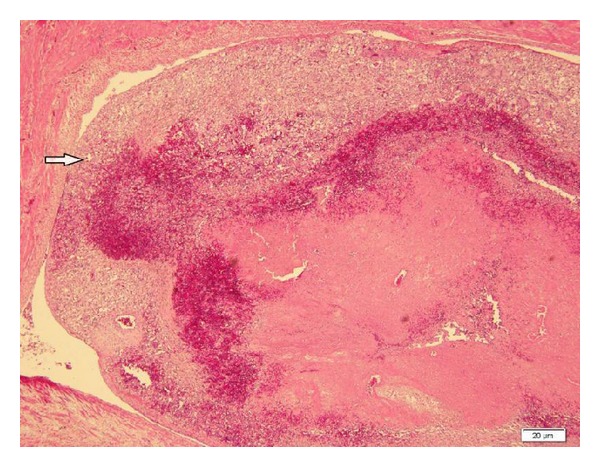
Embolus containing tumour cells (arrow) in the renal vein (H&E ×40).

**Figure 8:**
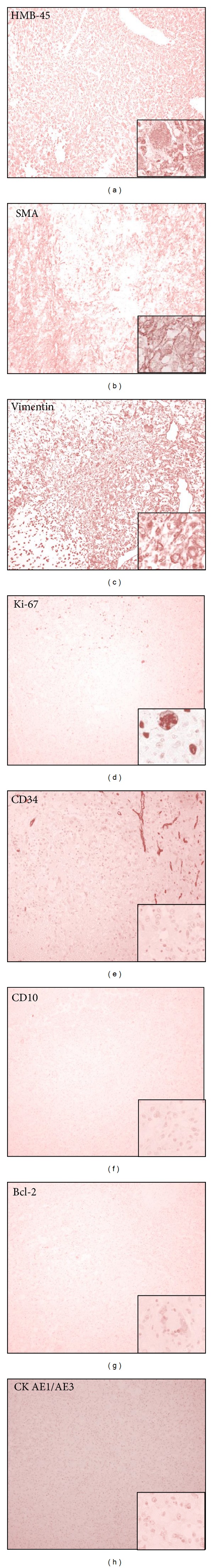
Immunohistochemical staining for the renal tumour. HMB-45, SMA, and Vimentin: positive for mononuclear and multinucleated giant cells of the tumour. CD34, CD10, Bcl-2, and CK AE1/AE3: negative. Ki-67 labelling index: 23%.

Microscopically, the tumour cells had a wide variety of appearances. Most of the cells were polygonal with abundant eosinophilic granular or clear cytoplasm. In some areas, the tumour contained spindle-shaped cells and multinucleated giant cells. The nuclei showed pleomorphism with prominent nucleoli and scattered mitoses (Figure 6). The cells were arranged in sheets or surrounded blood vessels. The tumour extended from the medulla to cortex and pelvis. In particular, emboli containing tumour cells appeared in the renal vein (Figure 7). Besides, the tumour had foci of haemorrhage in many areas. No evidence of lymphatic or capsular involvement could be seen. The immunohistochemical staining showed that mononuclear cells and multinucleated giant cells were strongly positive for HMB-45, SMA, and Vimentin and negative for CK AE1/AE3, CK7, EMA, CD10, CD34, and Bcl-2. The Ki-67 labelling index was about 23% (Figure 8). The final diagnosis of epithelioid angiomyolipoma was made. She continued to be treated at the hospital.  Table 1 summarizes the immunohistochemical results of both tumors.

## 3. Discussion

Usually, the diagnosis of solitary fibrous tumour was not made easily. At the first glance by H&E staining, many lesions with one or some features similar to solitary fibrous tumour should be thoroughly reviewed such as fibromatosis, fibrosarcoma, gastrointestinal stromal tumour (GIST), leiomyoma, leiomyosarcoma, and schwannoma. Compared with these tumours, the distinct features of solitary fibrous tumour are patternless arrangement of spindle cells, branching vessels, and parallel strands of collagen.

Immunohistochemically, CD34 is the most important marker for the diagnosis of solitary fibrous tumour.

90 to 95% of cases of solitary fibrous tumour are positive for CD34 [6]. In comparison with the other tumours of sigmoid colon, CD34 is negative for leiomyoma, leiomyosarcoma [7], fibromatosis, and fibrosarcoma [8] and positive for epithelioid gastrointestinal stromal tumour (80–90%) and glandular schwannoma [8]. The positivity of CD34 helped us to exclude leiomyoma, leiomyosarcoma, fibromatosis, and fibrosarcoma. We also performed immunohistochemical stain of C-kit and S100 for the tumour which did not show reactivity, excluding gastrointestinal stromal tumour and schwannoma, respectively. In the literature, Bcl-2 and SMA are variably positive for solitary fibrous tumour [6]. Desmin is negative for solitary fibrous tumour [8]. In summary, the positive reactivity of CD34, Bcl-2, and SMA and negative reactivity of C-kit, S100, and desmin of the tumour were compatible with solitary fibrous tumour.

The prognosis of solitary fibrous tumour was difficult to be assessed. Although majority of solitary fibrous tumours are benign, 10 to 15% of cases behave aggressively [6]. The criteria including increased cellularity, necrosis, and pleomorphism and increased mitotic rate (>4 per 10 high-power fields) could be applied for malignancy of solitary fibrous tumour of pleura and soft tissue [8]. In our case, increased cellularity and pleomorphism are significant in some areas of the tumour. Therefore, the tumour was interpreted as a malignant potential.

The second tumour in her right kidney which was interpreted as epithelioid angiomyolipoma also needed the differential diagnoses. Firstly, the component of epithelioid tumour cells with clear or eosinophilic granular cytoplasm and prominent nucleoli was found in clear cell renal cell carcinoma. However, the tumour cells were not arranged into typically acinar and alveolar pattern of clear cell renal cell carcinoma. The typical network of small thin walled blood vessels of clear cell renal cell carcinoma could not be found in the tumour. Secondly, the differential diagnosis of sarcomatoid renal cell carcinoma should be noted. The sarcomatoid component of sarcomatoid renal cell carcinoma is also composed of spindle, multinucleated giant cells, and usually high nuclear grade [9]. Besides, the group of mesenchymal tumours should be noted in this case.

The immunohistochemical staining is necessary for distinguishing epithelioid angiomyolipoma from clear cell renal cell carcinoma and sarcomatoid renal cell carcinoma. In the literature, 94% of clear cell renal cell carcinomas show positivity for CD10 [10]. Clear cell renal cell carcinomas are almost positive for CK AE1/AE3 and EMA [10]. In an immunohistochemical study of 18 cases of sarcomatoid renal cell carcinoma, 17 cases (94%) showed positive reactivity with CK AE1/AE3, and 9 cases (50%) showed positive reactivity with EMA [11]. The tumours cells did not react positively for all of CD10, CK AE1/AE3, and EMA. Besides, HMB-45 was important for the differential diagnoses. Renal cell carcinoma and most sarcomas are negative for HMB-45 [11]. Otherwise, epithelioid angiomyolipoma is a member of perivascular epithelioid cell tumours (PEComas) which commonly label for HMB-45, Vimentin, and smooth muscle markers and are negative for CD34 [12]. The negative activity of CK AE1/AE3, EMA, CD10, CK7, and CD34 and positive activity of HMB-45, Vimentin, and SMA of the tumour were consistent with epithelioid angiomyolipoma. The tumor is designated as pure epithelioid PEComas (so called “epithelioid angiomyolipoma”) of the kidney and confirms malignant potential [13].

Until now, there is no large study for Ki-67 expression of epithelioid angiomyolipoma. However, a study of Ooi et al. concluded that the Ki-67 was a useful marker which distinguishes the malignant epithelioid variant of angiomyolipoma from classic angiomyolipoma. In their study,all cases of epithelioid angiomyolipoma were strongly positive for Ki-67, while all cases of classic angiomyolipoma were completely negative [14]. Another report of 3 cases of angiomyolipoma mentioned that high Ki-67 expression was a feature of malignant epithelioid angiomyolipoma. On the contrary, benign angiomyolipomas were consistently negative for Ki-67 [15]. Besides, a study of 10 cases of epithelioid angiomyolipoma found that higher expression (positive) of Ki-67 indicates the presence of epithelioid angiomyolipoma and poor prognosis of patients [16]. In our case, along with solitary fibrous tumour with malignant potential and the appearance of tumour emboli, the Ki-67 labelling index of 23% indicated the malignancy for the renal tumour and poor prognosis.

We were also concerned about tuberous sclerosis because the patients of epithelioid angiomyolipoma usually have a history of this genetic disorders. However, except angiomyolipoma, the patient did not have the symptoms, family history, and history of the disease of tuberous sclerosis in other organs. The imaging findings of the patient in other organs were not suggestive of tuberous sclerosis.

The occurrence of both solitary fibrous tumour and angiomyolipoma was very rare in the literature. We just found a case report of solitary fibrous tumour of the adrenal gland with ipsilateral renal cell carcinoma and angiomyolipoma [17]. This patient was 48 years old and also a Japanese woman. In their report, photographic demonstration is limited to solitary fibrous tumour and not to the angiomyolipoma. Therefore, our report is the first on combining these tumours in the literature which demonstrates extensive immunohistochemical staining of both tumours. Other multiple primary tumours were very uncommon and might be related to several factors. According to Rosso et al., subsequent malignancies can initially result from intense clinical surveillance after the first tumour. They can occur later on as therapies for the first primary can induce carcinogenesis. Finally, they can also be due to shared risk factors, including environment, life styles, and inherited genes predisposing to higher susceptibility. However, the high fatality of several cancers hinders the possibility of observing subsequent events, even if their probability is sensibly increased [18]. The final diagnoses needed the combination of clinical information, medical imaging, and histopathology, especially the role of immunohistochemical staining. Because the patients had multiple primary tumours, the treatment was very complicated. Further studies of multiple primary tumours about the mechanism, risk factors, treatment, and prognosis are very necessary for solving these problems in these cases.

As the patient appears to lack any genetic background and due to the complete difference of immunophenotypes between these two tumours, we think the solitary fibrous tumour and the epithelioid angiomyolipoma are two unrelated separate tumours occurring in the same patient. Further genetic analysis may disclose the basic mechanism for this very unusual combination of two different tumours.

## Figures and Tables

**Table 1:** Immunohistochemical profile for the tumours.

Antibody	Tumour attaching to serosa of the sigmoid colon	Tumour of the right kidney
CD34	++	−
SMA	++	++
HMB-45	−	+++
Bcl-2	+	−
Ki-67 labelling index	10%	23%
S100	−	n.d
C-kit	−	n.d
Desmin	−	n.d
Vimentin	n.d	++
CK AE1/AE3	n.d	−
CK7	n.d	−
EMA	n.d	−
CD10	n.d	−

(−): negative; (+): <10%; (++): <50%; (+++): >50%; n.d: not done.
